# The Neonatal Immune System and Respiratory Pathogens

**DOI:** 10.3390/microorganisms11061597

**Published:** 2023-06-16

**Authors:** Colleen J. Sedney, Eric T. Harvill

**Affiliations:** Department of Infectious Diseases, College of Veterinary Medicine, University of Georgia, Athens, GA 30602, USA; colleen.sedney@uga.edu

**Keywords:** neonatal immunity, respiratory pathogens, mouse models

## Abstract

Neonates are more susceptible to some pathogens, particularly those that cause infection in the respiratory tract. This is often attributed to an incompletely developed immune system, but recent work demonstrates effective neonatal immune responses to some infection. The emerging view is that neonates have a distinctly different immune response that is well-adapted to deal with unique immunological challenges of the transition from a relatively sterile uterus to a microbe-rich world, tending to suppress potentially dangerous inflammatory responses. Problematically, few animal models allow a mechanistic examination of the roles and effects of various immune functions in this critical transition period. This limits our understanding of neonatal immunity, and therefore our ability to rationally design and develop vaccines and therapeutics to best protect newborns. This review summarizes what is known of the neonatal immune system, focusing on protection against respiratory pathogens and describes challenges of various animal models. Highlighting recent advances in the mouse model, we identify knowledge gaps to be addressed.

## 1. Introduction

Infants and neonates have long been considered to have an underdeveloped and ineffective immune system. This view emerged from the observed susceptibility to adverse outcomes of infection with particular pathogens that are more severe in infants than in adults. Infants also have distinct immune components and functions, which when evaluated against adult immune standards, were found to be defective in their activities [1,2]. For example, infants generate primarily anti-inflammatory TH2 responses that likely help maintain fetal–maternal tolerance and reduce the risk of damaging inflammatory responses against the many new commensal organisms encountered after birth [3,4]. This results in limited and/or delayed responses against some harmful pathogens, that can grow to cause more severe disease. However, increasing evidence suggests that newborns and neonates have a quite effective immune system that is very different from that of adults [5]. This includes populations of unique cells and subsets of immune cells that specifically make up the neonatal immune system. These neonatal cells also have differential expression of genes and factors which regulate the anti-inflammatory response towards pathogens. Pathogens which primarily cause severe disease in infants, such as *Bordetella pertussis*, influenza, and RSV, mainly infect and cause disease in the respiratory tract, suggesting a unique interaction between the neonatal pulmonary immune environment and these particular pathogens. However, we still have limited knowledge regarding how the differences between the neonatal and adult immune components affect their responses to pathogens, and how we can best utilize the unique capabilities of neonatal immune cells to develop targeted vaccines and treatments against neonate-specific pathogens.

The limited understanding of the neonatal immune system is in large part due to the lack of appropriate animal models to study the neonatal response to infection. Immunology has leaned heavily on the murine model, due to its low cost, reproducibility, and extensive library of immunological tools. Mice mature extremely rapidly, which facilitates studies of adult immunity, but greatly reduces the timeframe of the neonatal period from months in humans to only days in mice [6]. Although there is no discreet cutoff point, the general consensus of the field is that the neonatal stage of immune development in mice lasts only until ~7–10 days post birth. However, infections can progress over periods of days, weeks, or longer, so experimental infections initiated in neonates have ranged from 2 to 21 days post-birth (P2–P21), during which there are significant developmental shifts in the immune system. This lack of consistency with the use of neonatal murine models has resulted in some confusion that further frustrates a comprehensive understanding of the neonatal immune system.

In this review, we will discuss current knowledge of the development of the neonatal pulmonary immune environment, the rapid shifts in immune components, and striking differences in techniques used to assess them. We will also specifically address the literature regarding the neonatal murine immune response to *Bordetella pertussis*, RSV, and influenza, all of which primarily infect or cause disproportionately severe disease in infants. We will also highlight gaps in knowledge of the important differences between the neonatal and adult immune systems that limit optimal applications of vaccines and other approaches to protect highly sensitive newborns from respiratory disease, leading to recent rises in infant infections [7,8,9].

## 2. The Neonatal Immune System Is Composed of Unique Cells

**Figure 1:**
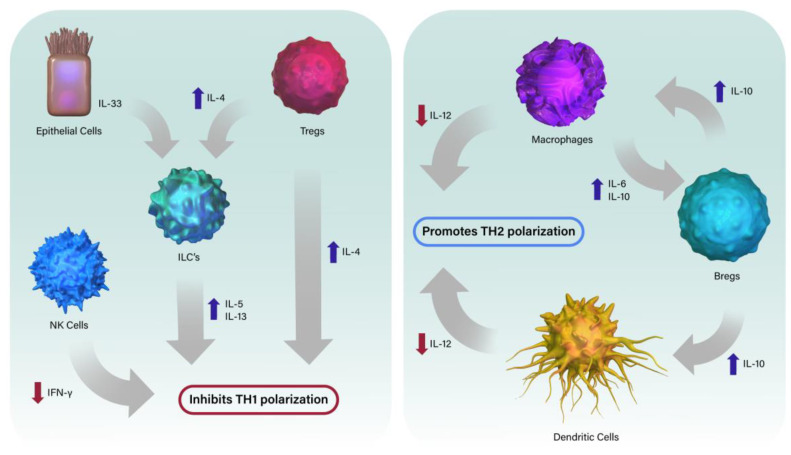
Schematic view of factors contributing to the inhibition of TH1 polarization and promotion of TH2 polarization in the neonatal pulmonary environment. Red arrows indicate decreased production and blue arrows indicate increased production. Illustration by Sofia Nahman, Educational Resources, University of Georgia College of Veterinary Medicine.

The immune system is a complex network of cells and signals which regulate the host’s response to self and foreign antigens. This delicate system requires substantial regulation to prevent severe damage to the host but is also balanced against the potential damage that could be inflicted if a response is not generated. Thus, responses are primarily dependent on the needs of the hosts and the nature of the signal. In the case of immune stimulation by pathogens, the primary goal of the immune system is to mediate clearance of the pathogen while minimizing damage inflicted to the host by the immune response itself. The adult immune system, which is better studied and understood than is the neonatal one, efficiently generates pro-inflammatory responses which mediate the efficient control of most pathogens. The neonatal immune system, however, is evolved to respond to the unique challenges of the rapid transition from the near-sterile womb to the microbe-rich world beyond. This suddenly introduces millions of new antigens for potential immune recognition and response, a seemingly impossible feat. Therefore, during this transition from near-sterile-fetal to microbe-rich neonatal environment, the immune system is evolved to respond to novel antigens primarily with anti-inflammatory TH2 responses to prevent unnecessary inflammation which can severely harm the infant (Figure 1). However, the increase in TH2-skewed responses does result in a delayed response to some actual pathogens, leading to the interpretation that the neonatal immune system is defective in general, compared to the adult immune system. However, the neonatal immune system can be very effective when the unique cellular components of the fetal/neonatal immune system are appropriately stimulated.

**Figure 2:**
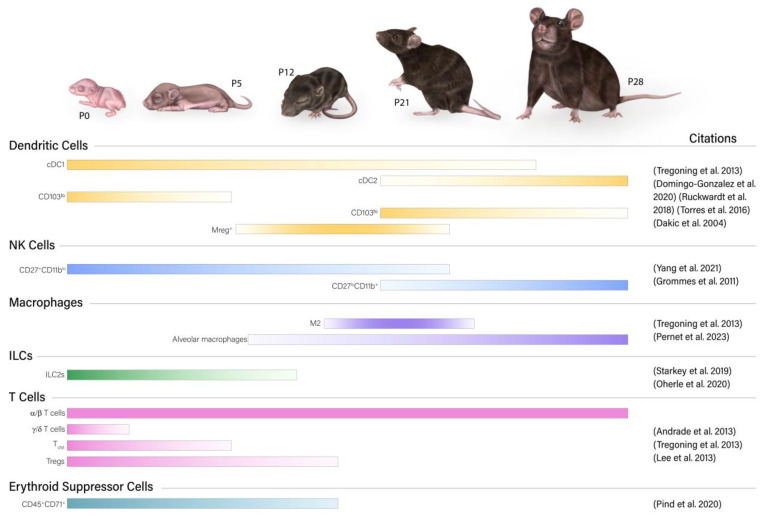
Relative frequency of immune cell types/subsets in the neonatal pulmonary immune system [10,11,12,13,14,15,16,17,18,19,20,21,22]. Illustration by Sofia Nahman, Educational Resources, University of Georgia College of Veterinary Medicine.

Understanding the differences between the adult and neonatal immune system can inform our ability to properly stimulate the neonatal immune system to make an effective response and lead to new approaches to specifically treat neonatal infections. This begins with the optimization of neonatal mouse models that can allow us to better understand how the neonatal immune system responds to various infections (Figure 2). Here, we describe current knowledge of the cellular components of the murine neonatal immune system, how they are regulated, and the wide range of ages which have been used and labeled as “neonatal”.

### 2.1. Innate Immune System

#### 2.1.1. Neutrophils

Neutrophils are white blood cells that rapidly respond to pathogens, particularly in the lungs. They release granules, reactive oxygen species (ROS), and ultimately neutrophil extracellular traps (NETs) that can trap and kill pathogens, but also can damage host cells. In adult mice, neutrophils develop from precursors in the bone marrow before migrating to the periphery [23]. Neutrophils are derived from the granulocyte-macrophage progenitor (GMP) and proliferative neutrophil precursor (preNeu), which expand under microbial stress before migrating to the periphery [23]. Their short half-life requires that they be consistently replenished in the periphery [23]. While few requirements are known for neutrophil development in murine models, iron regulatory protein (IRP) has been observed to be critical for neutrophil development and differentiation due to its role in iron homeostasis [24]. Together, the development and activities of neutrophils aid in the control and clearance of pathogens in the lungs; however, the specific roles of neutrophils in the neonatal lungs are unclear.

In adults, lung infection results in the rapid recruitment of neutrophils, via L-selectin-dependent migration [25]. Many studies have observed that neonatal neutrophils are deficient compared to those of adults, resulting in increased susceptibility to pathogens. One-week-old mice inoculated with RSV had less neutrophil recruitment to lungs compared to adults after seven days of infection (P14) [26]. After inoculation with *E. coli*, both P1 neonatal and adult mice were neutropenic at 1 day post-inoculation (dpi) (P2). However, the neonatal mice took longer to recover than did adults and the former did not experience the increases in neutrophils, G-CSF, and IL-6 production that were observed in adults [27]. Multiple studies suggests that this reduction in neutrophil recruitment may be affected by the production of IL-10 from B cells and dendritic cells. Compared to wildtype pups, TLR2-deficient pups inoculated with Group B *Streptococcus* at P2 were not susceptible to sepsis and did not produce increased amounts of IL-10 associated with damaging inflammation in wildtype pups at 24 h after infection (P3). Importantly, it was also observed that in the absence of TLR2 and the resulting IL-10, neonatal neutrophils were able to migrate to infected lungs and control infection [20]. This suggests that neonatal neutrophils have the inherent ability to migrate to infected tissues and stimulate appropriate immune responses, but that the anti-inflammatory regulation of the neonatal immune system modulates this ability.

There is significant evidence that neonatal neutrophils fundamentally have the same capabilities as adult neutrophils do to control pulmonary infections. Neonatal mice (P2) inoculated with influenza had a slightly slower accumulation of neutrophils to the lungs compared to adults at 7 dpi (P9) but had higher amounts of neutrophils than adults did at 10 dpi (P12). These neonatal neutrophils were also successful in infiltrating the alveolar spaces, demonstrating the ability of neonatal neutrophils to migrate to the site of infection [28]. Neonatal mice (P5) inoculated with a mutant of *Bordetella pertussis* lacking the pertussis toxin demonstrated neutrophil accumulation to the lungs as early as 2 h after inoculation, also demonstrating that neonatal neutrophils can respond very rapidly to the lungs. A subsequent increase in T cells in the lungs suggests that neonatal neutrophils can also efficiently recruit T cells. These responses, however, were not observed in P5 pups inoculated with wildtype *B. pertussis*, suggesting that pertussis toxin disrupts neutrophil accumulation in the lungs [29]. Similarly, a mutant of *E. coli* lacking Lpp was successfully controlled in mice and rats by P5–P9. This control required NADPH oxidase-generated ROS from neutrophils. However, this effect was not observed in wildtype *E. coli*, suggesting that Lpp suppresses neutrophil killing to promote disease in neonates [29]. This suggests that pathogens produce specific factors which disrupt neonatal neutrophils and prevent control of infection from neonatal lungs.

The immunosuppressive effects of pathogens on neonatal neutrophils, however, can be curtailed via pre-treatment with drugs that stimulate the neonatal immune system. Pretreating neonatal mice (P5–P7) with alum 24 h before the induction of polymicrobial sepsis improved phagocytosis by peritoneal neutrophils resulting in decreased pathogen numbers by 24 h post-challenge (P6–P8). Alum pretreatment also increased numbers of NET-positive neutrophils and the expression of co-stimulatory molecules (CD80 and CD86) [30]. This suggests that peripheral neonatal neutrophils can successfully respond to microbial infection but require differential stimulation to generate a protective response. This process is likely applicable to neutrophils in neonatal murine lungs; however, further work is required for definitive results. Similarly, when treated with Memantine and inoculated with *P. aeruginosa*, P7–P8 Sprague Dawley (SD) rats had significantly reduced bacterial loads in the lungs, blood, liver, and spleen, accompanied by the inhibition of IL-6 production [31]. This evidence suggests that stimulation of neonatal neutrophils can result in increased anti-microbial efficiency and successful control.

Though neutrophil activity is typically associated with a protective response, this activity can also cause tissue damage, threatening the health of neonates. The primarily anti-microbial activities of neutrophils include the release of granules which results in bacterial killing, regulation of cytokine signaling, and stimulation of NETs and ROS by neutrophils in an autocrine/paracrine manner. These anti-microbial functions, however, can cause serious damage in neonatal lungs and can exacerbate infections. This is due to the fact that NETs can cause non-specific inflammation which allows pathogens to escape the immune response and generate severe pulmonary and systemic disease [32,33,34]. It has also been observed that NETosis can be regulated via cytokines, with IFN-γ signaling limiting NETosis and resulting in better outcomes in pups with viral bronchitis [35]. The production of ROS by neutrophils has also been implicated in acute lung injury of neonatal mice [15,16]. Therefore, while neonatal neutrophils have the ability to respond similarly to adult neutrophils, the specific challenges of neonates require these activities to be modulated to prevent unnecessary damage, an opportunity some pathogens may take advantage of.

#### 2.1.2. NK Cells

Natural killer (NK) cells are lymphocytes of the innate immune system that derive from the same precursors as T and B cells and are also a member of the Group 1 innate lymphoid cells (see below). They function to release granules to kill pathogens and signal the immune system. NK cells are first detected in the lungs as immature CD27^+^CD11b^lo^ cells. Mature CD27^lo^CD11b^+^ NK cells first appear in the lungs at 3 weeks and are the primary NK cell population by 8 weeks [36,37]. Mice lacking FcRn have low levels of immature NK cells, indicating that FcRn plays a role in their development. These NK cells also express lower levels of CD107a, which suggests reduced degranulation, and produced smaller amounts of IFN-γ, though their cytotoxicity was similar to that of mature adult NK cells [38]. However, there is also evidence that neonatal NK cells can function to control pulmonary infections. P2 pups inoculated with *Chlamydia muridarum* had a significant accumulation of NK cells in the lungs by 3 dpi (P5) accompanied by decreased bacteria in the lung [39]. Additionally, the ablation of erythroid suppressor cells in P6 pups inoculated with *B. pertussis* resulted in increased NK cell accumulation in the lungs and increased protection against *B. pertussis* by 4 dpi (P10) [40]. This evidence suggests that there is a protective role of neonatal NK cells in controlling infections in the neonatal lung.

In addition to producing granules to mediate microbe killing, NK cells are also a major producer of IFN-γ. Though neonates primarily rely on the anti-inflammatory TH2 response, IFN-γ may play a role in protecting neonates from infections. IFN-γ-deficient mice inoculated at P2 with the measles virus suffered increased lethality compared to wildtype pups at 6 dpi (P8) [41]. Additionally, IFN-γ can play a unique role in the neonatal immune response by preventing ROS and NETosis by neutrophils, thereby preventing inflammation of the lungs in neonates [35]. Conversely, IFN-γ can also suppress production of antibodies [10,11].

#### 2.1.3. Dendritic Cells

Dendritic cells are antigen-presenting cells which can induce T cell activation via phagocytosis of pathogens and the presentation of antigens. There are two primary categories of DCs, plasmacytoid (pDC) and conventional (cDC). pDCs specialize in secreting type I interferons while cDCs (further separated into groups 1 and 2) specialize in antigen presentation to activate TH1 and TH17 responses. Though most DCs fall into these categories, there are numerous additional subsets which exist, particularly in neonates during development. Single-cell sequencing analyses of C57Bl/6 pups at various stages of development have revealed that neonatal murine lungs contain cDCs, as well as an additional subset characterized by the expression of melanoregulin (*Mreg*). cDC1 was the most abundant subset in the lungs from P1–7, with cDC1 and cDC2 being in the lungs in equal amounts by P21. Additionally, the expression of *Itgae* and *Cd209a* by neonatal DCs suggests that they can induce T cell immunity upon birth to generate an effective immune response. Interestingly, the *mreg*-expressing DCs were not detected prior to birth but were present in high numbers at P7 before decreasing at P21. This suggests a unique migratory DC subset in neonatal murine lungs [11]. Similarly, two subsets of migratory CD103^+^ DCs were observed in the lungs of neonatal mice inoculated at P7 with RSV. These subsets (CD103^lo^ and CD103^hi^) were observed starting at 1 dpi (P8) and during infection with influenza. These two subsets were also found to have distinct functional characteristics, including the presence of co-stimulatory molecules and ability to stimulate specific responses. Though the CD103^hi^ DC subset was found to have superior function and increased amounts of co-stimulatory molecules, CD103^lo^ DCs were more prominent in neonatal lungs [12]. The different functions and characteristics of these DC subsets may contribute to the decreased TH1 activation of neonatal T cells.

DC populations in the neonatal thymus are similar to those of adults by P7, though they proved to be less efficient at antigen processing and presentation at this stage [42]. After egress from the thymus, they shift greatly during mouse development. P7 neonatal mice had low levels of GM-CSF compared to naïve adults, which is believed to limit the development of CD103^+^ DCs. However, P7 neonatal mice inoculated with RSV produced a CD103^+^ DC response at 7 dpi (P14) [12,42]. CD11b^+^ DC populations were low at P6 but increased greatly during the first 3 weeks of life. Additionally, DCs from P7 pups were unable to transport antigens from the lung to the lymph nodes at the same efficiency as adults were able to [42]. It was also observed that neonatal CD103^+^ DCs stimulate CD8^+^ T cells differently than do adult CD103^+^ DCs. While neonatal DCs can present antigens at the same efficiency as adult DCs can, they have decreased expression of the costimulatory molecules CD28, CD80, and CD86, resulting in a failure to stimulate CD8^+^ T cell proliferation and a limited CD8^+^ T cell response [12,13,42]. Neonatal lungs had more cDC1 subsets; however, these proportions shift during development, with adults having more cDC2s in the lungs [14,42,43]. Surprisingly, neonatal DCs also have differential cytokine production, with DCs isolated from the spleen displaying slightly increased production of IL-12p40, an important inducer of TH1 responses [13,14]. This was accompanied by increased production of IL-10, further repressing activation of CD8^+^ T cell responses [13].

One of the major issues with the neonatal immune system is the apparent inability to generate TH1 responses, as these are considered the most effective against some pathogens. The development of this response requires early exposure of immune cells to IFN-γ followed by priming with IL-12 [44]. However, the development of this response is impeded by the production and actions of IL-10, of which neonatal immune cells are major producers. It has been observed that neonatal DCs have the capacity to generate TH1 responses by producing IL-12 and stimulating T cells; however, this process is affected by IL-10 production, particularly from neonatal B cells [44]. The shift in the ability to produce IL-12 and generate a TH1 response occurs at P6 in mice when the DC populations increase and subsets shift away from the neonatal pattern and toward the adult-like one, causing a shift in the response to be primarily TH1 [45].

#### 2.1.4. Macrophages

Macrophages are immune cells that phagocytose pathogens and are derived from the same progenitor as neutrophils are [46]. Single-cell sequencing identified unique clusters of macrophages that shift from fetal development to post-birth [11]. Prior to birth, the primary cluster of macrophages (Mac I) in the lung are highly proliferative and localize to small vessels, likely to promote growth and remodeling. A new cluster (Mac II) arises the day after birth at P1 with possible roles in immune regulation and tissue remodeling [11]. This subset is of particular importance as it is detected at the beginning stages of postnatal alveolarization, a process which begins after birth and peaks at P39 [47,48]. Another cluster of alveolar macrophages (Mac III) was also observed at P1 and P7. These clusters of macrophages (Mac I, II, and III) expressed genes that promote bacterial killing but suppress inflammation, likely contributing to the anti-inflammatory response in the lungs at P1 and P7. Surprisingly, macrophages with proinflammatory signatures (Mac IV), likely contributing to cytokine production and leukocyte chemotaxis, were also identified in P1 and P7 mice. Domingo-Gonzalez et al. suggested that the Mac II cluster of macrophages are a transitory subset, due to the overlap of expression with Mac I and Mac III clusters [11]. This work displays the dramatic shifts in macrophage populations and phenotypes that occur during neonatal lung development. Additionally, one of the most important subsets in the neonatal lung are alveolar macrophages due to their anti-inflammatory tendency to prevent damaging inflammation. Fetal monocytes develop into alveolar macrophages by P7 and persist for approximately 3 months [49]. Differentiation of monocytes into alveolar macrophages was also aided by lung basophils [50]. The persistence and maintenance of alveolar macrophages requires GM-CSF and neonatal neutrophil-derived 12-hete [17,49]. An additional subset of macrophages which exist in the lungs are M2 macrophages. These are in lungs at highest amount from P14–P21 and have roles in tissue remodeling and immunosuppression [51].

Neonatal macrophages are generally believed to have similar functions and capabilities as adult macrophages. However, neonatal macrophages adhere, spread, and phagocytose in a CR3-dependent manner while adult macrophages complete the same activity in a CR3-independent manner [52]. Similar to other immune cells, neonatal macrophage functions are often affected and modulated by the neonatal pulmonary environment. The production of IL-6 and IL-10, and lack of production of IL-12, by macrophages prevents the production of IL-1β and TNF-α via stimulation with LPS [53]. The reduction of IL-10 allowed for the increased activation of neonatal macrophages [54]. The regulation of macrophages by IL-10 can also be modulated via the pretreatment of neonatal mice with alum, which increased phagocytosis and costimulatory markers on neonatal macrophages [30]. The response of neonatal alveolar macrophages in pups inoculated with RSV was also improved by the addition of IFN-γ [55]. Thereby, neonatal macrophages can play important roles in protecting neonates from pulmonary infections; however, their function can be greatly improved via TH1 skewing.

#### 2.1.5. Innate Lymphoid Cells

Innate lymphoid cells (ILCs) are immune cells with important roles in cytokine production and similar functions to T cells but lack the ability to display antigens and subsequently activate B cells. There are three groups of ILCs that are differentiated by the cytokines they produce. Group 1 ILCs (ILC1s), which includes NK cells, primarily produce IFN-γ and TNF-α and are involved in immunity to bacteria, viruses, and cancer, while Group 3 ILCs (ILC3s) produce IL-22, IL-17A, and IFN-γ and are involved in immunity to bacteria, chronic inflammation, and lymphoid development. While ILC1s and ILC2s can be recruited to the lungs, Group 2 ILCs (ILC2s) are naturally resident in the lungs [18,56]. These cells make IL-5 and IL-17 in response to stimulation with IL-33 and IL-25. In neonatal IL-33-deficient mice, ILC2s are still observed; however, they are not activated [57]. Pulmonary ILCs descend from ILC precursors that populate a niche defined by fibroblasts in the developing lung. The fibroblasts make insulin-like factor 1 and this instructs the expansion and maturation of pulmonary ILC precursors. Depleting IGF-1 prevented ILC3 development which led to increased susceptibility of neonatal mice to pneumonia [19]. Lung ILC2s were found starting at P4 and peaked at P14 then decreased as the lungs matured [58]. ILC development is also dependent on the transcription factor RORα. In P12 lungs, three populations of ILCs were found. One was a progenitor population similar to that in adults and the two others differentially produce TH2 cytokines and amphiregulin. Together, these subsets have distinct proinflammatory and tissue-repairing subsets [59]. ILC2s increase after birth and peak at P10, where they are found at three-fold higher levels than those in adult lungs. At P11, ILC2s uniquely express IL-5 and IL-13, proliferate via IL-33 signaling, and promote TH2 immunity [60].

The production of cytokines from ILCs generates and maintains the unique neonatal immune environment. Specifically, the production of IL-13 by neonatal ILCs maintains the M2 status of macrophages [61]. An important mediator of this response is IL-33, produced by epithelial cells and associated with alveolarization and tissue remodeling of the lungs, along with acute TH2 responses [47,60]. However, ILCs can also induce damaging inflammation. P5 pups inoculated with RSV had increased IL-33 expression and increase in ILC2 numbers in lungs at 1 dpi (P6), a response which was not observed in adults. This response also led to RSV immunopathogenesis and was inhibited by IL-33 depletion [47,62]. Additionally, P6 neonatal mice with rhinovirus demonstrated increased IL-13 and IL-25 as early as 1 dpi (P7) with suppressed IFN-γ, IL-12p40, and TNF-α expression while Group 2 ILCs populations making IL-33 were expanded. This response was attenuated by IL-25-neutralizing antibodies, implicating IL-25 as an additional mediator of ILC activity [63].

#### 2.1.6. Myeloid-Derived Suppressor Cells

Myeloid-derived suppressor cells (MDSCs) are similar to neutrophils and monocytes; however, they express potent immunosuppressive abilities, primarily of T cell responses. They are activated by T cell-derived IFN-γ but then suppress T cells via the expression of iNOS and arginase 1, which generate NO and urea, respectively [64,65]. While they suppress α/β T cells, they also promote the development of Tregs in an IL-10-dependent manner [66]. Neonatal mice specifically have a large transitory population of MDSCs in the lungs in the first few weeks of life, while adult mice have reduced populations throughout life. Their potent antimicrobial activities aid in the protection of neonates from infection in these first few weeks [65]. While MDSCs support and protect the neonatal immune system such that it reduces inflammation, they can also then cause reduced responses to pathogens.

### 2.2. Adaptive Immune System

#### 2.2.1. T Cells

T cells are lymphocytes that are developed in the thymus and participate in immune responses via the regulation and production of cytokines which mediate the responses of other cells. T cells can be activated by antigen-presenting cells (APCs) that present their specific antigen. After activation, T cells then expand and produce cytokines to promote additional responses from lymphocytes. There are two main types of T cells that are defined by the type of T cell receptor (TCR), α/β or γ/δ, that they express. While γ/δ T cells are the first to exit the thymus during neonatal development, most T cells in the neonatal lung are α/β until P21 [11]. γ/δ T cells mediate responses to influenza and generate TH2 responses in the lung [67]. T cells also have functions in cytokine production, B cell activation, and phagocytosis. T cells with specific functions are classified into different subsets which mediate different responses. Most neonatal T cells are activated to generate TH2 responses, defined by the production of IL-4 and IL-13. These cytokines promote the differentiation of B cells to produce IgE antibodies and M2 status of macrophages, and repress the production of IFN-γ. To generate these responses, T cells need to be activated via the presentation of their specific antigen by antigen-presenting cells. Once activated, T cells increase production of cytokines and often migrate to sites of infection. While neonatal T cells can be successfully activated by DCs, the lack of costimulatory molecules on DCs reduces this efficiency [13,42]. Additionally, CD62L^+^ T cells which can migrate to sites of infection are in low numbers in neonatal lungs [68].

While traditional α/β T cells are prominent in adult lungs, neonatal lungs have specific subsets, notably virtual memory T cells (T_VM_), which are classified by their naïve status with memory-like markers that allow for rapid responses. Additionally, they can be activated independently of antigens, instead rapidly expanding after cytokine signaling [21,69]. These are observed in highest amount at P8 and are greatly reduced by P10, suggesting they are a significant component of the neonatal immune system [70]. Neonatal mice also have populations of regulatory T cells (Tregs) which are primarily anti-inflammatory and produce TH2 cytokines and responses. Neonates also have substantial populations of Tregs, which have been observed to regulate iBALT [71]. This is in contrast to Tregs in adult lungs, which have been observed to interfere with BALT development [72]. Importantly, P2 neonatal T cells were more likely to develop into Tregs than adult T cells were, but this ability diminished by P14 [73]. Additionally, infection of P2 neonatal mice with influenza required Tregs for clearance at 6–10 dpi (P8–P12) [74]. Tregs were also required for responses to LPS by P2–P5 mice by 3 dpi (P5–P8), though this shifted after the neonatal stage (P12–P20) [75]. Additionally, beginning at P14, Tregs require PG-L1 for development, indicating a shift in immune system maturation [76].

T cell activity, specifically activation and expansion, can also be significantly affected by innate immune cells. MDSCs (discussed above) have a primary role in T cell suppression [65]. Despite this, it was observed that P5 mice inoculated with a mutant of *B. pertussis* had an influx of neutrophils into the lungs, followed by T cells at 1 dpi (P6) [70]. However, it has also been observed that T cells are defective in migrating to neonatal alveolar spaces in lungs [28]. This evidence suggests an important role of neutrophils in mediating subsequent T cell responses in neonatal lungs. Additionally, the depletion of alveolar macrophages can significantly reduce neonatal T cell populations in the lungs [65,77].

#### 2.2.2. B Cells

B cells are lymphocytes developed in the bone marrow that rearrange immunoglobulin genes to produce a surface antibody, can present peptides from recognized antigens, and with or without T cell help, can develop into different types of antibody-secreting plasma cells. While neonatal mice have high B cell numbers in the lungs, they do not proliferate like they do in adults [31]. In addition to having lower numbers of B cells than those in adult mice, the composition of the B cell subsets in neonates differs greatly [22]. One particular subset in neonatal lungs is that of regulatory B cells (Bregs), differentiated by their production of IL-10. They colonize the lungs in the first week of life but are found in small numbers in adult lungs. The production of IL-10 has numerous effects on the neonatal immune system, including dysregulated neutrophil migration, T cell activation, and macrophage activation [13,20,30,44,78]. This can limit the neonatal immune response to pathogens. In fact, pups inoculated with RSV demonstrated IFN-I production by AM, but this process was then repressed by IL-10 from Bregs [78].

#### 2.2.3. Erythroid Suppressor Cells

CD45^+^CD71^+^ erythroid cells (CECs) are generated in the bone marrow and are strong regulators of the neonatal immune response. Their main functions include suppression of T cell immunity and production of ROS [79,80]. Neonatal mice (P3) had significantly more expansion of CECs than adult mice had and this resulted in the increased suppression of T cell activation [78]. P6 neonatal mice were replete with CD71^+^ CECs and highly susceptible to *B. pertussis*, resulting in increased mortality by 8 dpi (P14) [79]. The depletion of CECs in neonates resulted in decreased susceptibility to *B. pertussis* in the lungs. It was also observed that the impaired phagocytic ability of CD11b^+^ cells contributed to increased susceptibility and was mediated by CEC-derived arginase II [40,79,80,81]. P6 mice were also found to be susceptible to *L. monocytogenes* and *E. coli* infections in the lungs. Inoculation of mice at P15, however, resulted in 100-fold less bacteria in the lungs than that in those inoculated at P6. These older mice also had 60% fewer CECs than the younger mice had. This suggests a relationship between the relative abundance of CECs in neonatal/juvenile mice and the ability to control bacterial infections [81].

## 3. Immune Proteins in Neonatal Lungs

### 3.1. Antibodies

In order to develop into antibody-secreting plasma cells, B cells are typically activated by antigens and stimulated by helper T cells. This process often takes place in secondary lymphoid organs, most notably the lymph nodes. However, naïve neonatal mice have poorly organized lymph nodes and low T and B cell numbers, resulting in low antibody titers [82]. Antibodies are produced as various isotypes (IgM, IgD, IgE, IgA, and IgG) which serve different roles in different types and stages of immune responses. The first isotype produced is IgM, followed by a process called class switching in which the isotype heavy chain is changed based on immune signals. While neonatal B cells can make antigen-specific antibodies and produce similar amounts of IgM to those that adults do, neonatal B cells have low AID expression [82]. This enzyme is responsible for isotype and class switching, and thus neonatal mice have reduced IgG titers compared to adults. However, early exposure to adjuvants or pretreatment with IFN-α can induce improved antibody responses [82,83]. Vaccination with Titermax Gold adjuvants at the day of birth (P0) has been observed to enhance the maturation of neonatal lymph nodes as early as at P1. These mice also generated antigen-specific IgG within 21 days after vaccination (P21) [82]. Additionally, P4 mice pretreated with IFN-α and then inoculated with RSV at P5 had increased B cell numbers and B cell activation at 14 dpi (P19). This treatment also increased RSV-specific IgA at 7 dpi (P12) and induced the expression of B cell-activating factor (BAFF) in the NALT [83]. Thereby, neonatal mice are limited in their ability to naturally produce antibodies but also demonstrate an ability to produce antibodies and class switching upon stimulation.

IgG and other antibody isotypes play important roles in the neonatal response to pathogens, and much of the early neonatal IgG titer is transferred from the mother. Multiple studies have demonstrated the importance of maternal antibody transfer, a process mediated by a specialized receptor, the neonatal Fc receptor (FcRn). P6–P7 neonatal mice are protected from *E. coli* infection by maternal IgG obtained via the placenta or breast milk. This transfer of antibodies and protection required FcRn to transfer IgG from a dam’s milk to the serum of neonates [84]. Maternal antibodies also protected P7 and P14 pups from lethal challenge with herpes simplex virus (HSV). Importantly, optimal protection required the transfer of antibodies via the milk and placenta. Additionally, pups inoculated at P7 and assessed at 14 dpi (P21) specifically required antibodies that activate Fcγ receptor 4 (FcγR4), suggesting an important role for antibody-dependent cellular cytotoxicity (ADCC) [85]. However, this protection conferred by maternal antibodies can also impede the primary generation of antibodies by neonates. Maternal antibodies can prevent B cells at neonatal germinal centers from developing into plasma cells and memory B cells, as well as prevent the isotype switching of antibodies. They also limit T follicular helper T cell expansion, thus affecting B cell activation and differentiation into plasma cells [86]. Therefore, maternal antibodies provide critical protection to newborns, but can also impede the neonatal primary development of antibodies.

As previously discussed, there is increasing evidence suggesting that specific pathogens may take advantage of the differences of the neonatal immune system to cause serious disease. Thus, there is considerable interest in protecting neonates via vaccination and preventative treatment. Maternal vaccination against *Candida albicans* protected P3 pups inoculated with the same fungus at 3 dpi (P6). The transplacental transfer of antibodies was found to be critical for the protection and adoptive transfer of vaccinated maternal serum similarly protected pups [87]. Additionally, pups born to an antigen-vaccinated mother had high titers of antigen-specific antibodies on the day of birth (P0) [82]. Experiments have also been conducted to assess the feasibility of the primary vaccination of neonatal pups. Martin Aispuro et al. vaccinated P7 pups with an outer membrane from *Bordetella pertussis*, followed by a booster at P21 and challenge with *B. pertussis* at P35. At 7 dpi, vaccinated pups had greatly reduced bacterial numbers in the lungs compared to unvaccinated mice. Additionally, pups vaccinated with the outer membrane vaccine had high titers of anti-pertussis toxin antibodies, an important marker of immunity, 14 days after the last booster (P35) [88]. However, with the immunization and inoculation course completed at 42 days post-birth, the information gained about the neonatal response is limited. Similarly, Noh et al. vaccinated P7 pups with a RSV glycoprotein core fragment followed by a booster at P21. At P35, while mice had RSV-specific antibodies, this humoral response was determined to be Th2-skewed due to the lower IgG1/IgG2 ratio observed compared to that in RSV-convalescent mice. When inoculated 4–5 weeks after the booster (P49–P56), immunized mice had a significant reduction in viral titers at 4 dpi (P53–P60) [89]. This suggests that responses developed in the neonatal period can have effects that extend into the juvenile and adult period. However, additional experiments originating and terminating in the neonatal period are required to better understand these effects. To this end, mice injected with vaccinia virus at P7–P8 had strong polyclonal B cell activation and IgG secretion 6 days after infection (P13–P14). However, when injected with alum to test responses to hapten carrier conjugates, P8 mice did not generate antibodies by 6 dpi (P14). By 10 dpi (P18), antibodies were detected, though at significantly lower titers than those observed in adults and the neonatal B cells were defective in their ability to differentiate into IgM- and IgG-secreting plasma cells [90]. P1 pups administered monoclonal antibodies specific for the SpA protein of *Streptococcus aureus* and then challenged 24 h later to assess the efficacy of antibody treatment (P2) survived better than control pups did, suggesting antibodies alone are able to protect neonates [91]. This evidence illustrates the limited ability of neonatal B cells to produce effective antibody titers, but also the important role of IgG in neonatal responses.

### 3.2. Complement System

The complement system is a cascade of enzymatic components which rapidly amplify the highly localized release of anti-microbial and pro-inflammatory signals that enhance the functions of antibodies and phagocytes. However, few studies have assessed the role of the complement system in neonatal mice in the control of pathogens. The critical C3 protein, central to the complement cascade, is not transferred from the mother, but produced by the infant and is present at low levels in their serum [92]. Importantly, these low levels do not rapidly increase upon neonatal stimulation with tetanus toxoid, as they do in adults. Neonatal macrophages also have a limited capacity to synthesize C3 upon LPS exposure [93]. Despite the low levels of complement observed in neonatal mice, this system can play an important role in responses to infection. In experiments by Wessels et al., C3^−/−^ and C4^−/−^ dams were vaccinated against Group B *Streptococcus* and the resulting pups were inoculated with Group B *Streptococcus* at P2. While pups born to C4^−/−^ dams were able to control infection, a majority of the C3^−/−^ pups succumbed to infection by 2 dpi (P4). This suggests that C3 is required for the generation of appropriate responses against bacterial infection [94]. These results suggest that the complement system may play an important, though poorly understood, role in mediating the neonatal response to pathogens.

## 4. The Neonatal Immune System Is Susceptible to Particular Pathogens

### 4.1. Bordetella Pertussis

*B. pertussis* is a Gram-negative respiratory pathogen and the causative agent of “whooping cough”. This bacterium is highly infectious via respiratory droplets, efficiently infects and grows in the upper respiratory tract and may move to the lower respiratory tract where it causes more severe symptoms, progressing from a runny nose, to a sore throat, and intense coughing [95]. While adults typically either experience asymptotic infections or mild, cold-like symptoms, infants are more susceptible to deeper respiratory infections with more serious symptoms, such as an intense paroxysmal cough, leukocytosis, and pneumonia [96]. Briefly, ~33% of *B. pertussis* infections in children under 1 year old require hospitalization and infants under 6 months old make up ~90% of deaths [97,98]. While vaccines against *B. pertussis* are available and widely utilized, they are not administered to infants under 2 months old [99]. These vaccines in adults also do not prevent asymptomatic nasal carriage and transmission, and therefore, infants are often infected by asymptomatic caregivers [100,101]. This likely stems from the vaccines being developed for and evaluated in children older than the most susceptible group (>6 months) [102]. Additionally, the efficacy of current and potential vaccines are often tested with adult animal models [103,104,105,106]. In addition to imperfect vaccines, antibiotic treatments have limited effect after the presentation of diagnostic symptoms [107]. Therefore, more information on the effects of *B. pertussis* on the neonatal immune system is required to develop the most appropriate treatments and vaccines.

**Figure 3:**
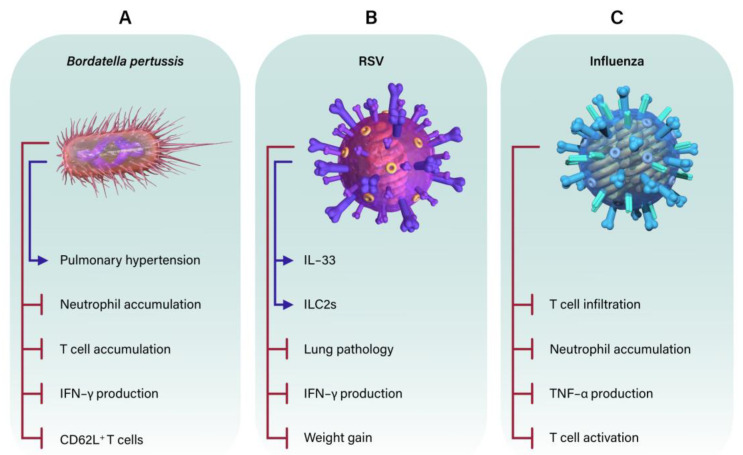
Effects of *Bordetella pertussis* (**A**), respiratory syncytial virus (RSV) (**B**), and influenza (**C**) on the neonatal pulmonary response. Blue arrows represent promoted/increased production. Red lines represent decreased/inhibited production. Illustration by Sofia Nahman, Educational Resources, University of Georgia College of Veterinary Medicine.

Some experiments have used neonatal mice to assess the specific response against *B. pertussis* [68,70,88]. These experiments suggest that, like human infants, neonatal mice are more susceptible to *B. pertussis* than adult mice are. Neonatal mice and humans fail to control *B. pertussis* infection and suffer increased mortality and lung inflammation [68,70]. Neonatal mice also develop pulmonary hypertension, one of the primary causes of death of human infants with *B. pertussis* [108,109]. Recent work suggests that this increased susceptibility is due to the dysregulated activity of neonatal immune cells caused by bacterial factors. For example, the reduction in CEC populations in P7 neonates resulted in reduced susceptibility to *B. pertussis* (Figure 3A) [40]. A possible mechanism for this increased susceptibility is the production of arginase II, which impairs the phagocytic ability of CD11b^+^ cells [40]. Neonatal mice also have a T cell subset of cells called virtual memory T cells (T_VM_) (see above) [70,110]. It was observed that as mice aged, their T_VM_ populations and susceptibility to *B. pertussis* greatly decreased [70]. Experiments aimed at assessing neonatal vaccination against *B. pertussis* utilized P7 neonatal mice immunized with an outer membrane protein. Vaccination greatly reduced bacterial numbers in the lungs and elicited high levels of anti-pertussis toxin antibodies [86]. These results suggest anti-pertussis toxin antibodies can provide some level of protection to neonates.

The pertussis toxin (PTx) is an AB_5_ toxin which disrupts G-protein-coupled receptors and subsequent signaling cascades [111,112]. Currently, the primary mechanism with which to assess clinical protection against *B. pertussis* is anti-PTx antibody titers, though the true protection provided by these titers is debated [113,114,115]. The primary effects of PTx on *B. pertussis* disease in adult mice include delayed lymphocyte accumulation to the site of infection, the suppression of serum antibody responses, and airway inflammation, in addition to important roles in early colonization [116,117,118,119,120,121]. The largest contribution of PTx to *B. pertussis* infections in adults was observed by Kirimanjeswara et al., in which transferred antibodies rapidly cleared a mutant of *B. pertussis* lacking PTx (*B. pertussis∆ptx*) but not the wildtype *B. pertussis.* The ~10,000-fold reduction observed with the PTx mutant indicates that PTx effectively blocks rapid antibody-mediated clearance [119], allowing *B. pertussis* to persist in immune hosts. More recently, PTx was shown to have greater effects on *B. pertussis* infections in neonatal mice than those in adults [68,70,118]. P7 mice inoculated with *B. pertussis∆ptx* had a ~100-fold reduction in bacterial numbers in the lungs at 14 dpi (P21) compared to wildtype *B. pertussis* [68]. In a similar model, P7 neonatal mice inoculated with wildtype *B. pertussis* had symptoms of pulmonary hypertension, while those inoculated with *B. pertussis∆ptx* lacked these symptoms [108]. However, an even greater effect was observed in mice inoculated at P5 and assessed at P8, a period more completely dominated by neonatal immunity, in which *B. pertussis∆ptx* had a >10,000-fold reduction in bacterial numbers compared to wildtype *B. pertussis.* This effect, much greater than that observed in adult mice, was accompanied by neutrophil accumulation as early as 2 h post inoculation with *B. pertussis∆ptx*, followed by increased T cell populations at 1 dpi (P6). Inoculation with the wildtype *B. pertussis*, however, did not cause any increases in immune cell populations until 3 dpi (P8). Importantly, these younger mice displayed a more purely neonatal immune environment, with substantial populations of T_VM_ at P8, and modeled the increased susceptibility of human infants to *B. pertussis* [70]. These experiments indicate that the neonatal immune system could effectively control *B. pertussis* infections, but some key functions are blocked by PTx. Further study of these will likely reveal important immune protection mechanisms in neonates.

### 4.2. Respiratory Syncytial Virus

Respiratory syncytial virus (RSV) is a member of the *Pneumoviridae* genus that is transmitted via respiratory droplets or fomites. Infection begins in the upper respiratory tract but then spreads to the lower respiratory tract via viral infection of airway epithelial cells. This most importantly can lead to bronchitis and pneumonia, though most common symptoms include coughing, a runny nose, and fever [122]. However, infection with RSV can have more long-term effects, such as the development of asthma. Nearly all children have been RSV-infected by age 2 and the most serious disease is observed in children under age 1 [123]. It is also a leading cause of infant hospitalization. Despite this, there are currently few approved vaccines to prevent infection, with 121 active clinical trials worldwide as of 2019, with the first United States RSV vaccine being approved for adults over 60 years in 2023 [124,125]. Due to the extraordinary burden on neonates, several of these vaccine candidates, and new work, are focused on understanding RSV in the neonatal immune system [124].

Due to the lack of current vaccine and treatments, research into RSV is of upmost importance. RSV viral replication was found to be similar in both neonatal and adult mice. However, P7 mice were found to have low levels of IFN-γ, CCL2, CCL3, CXCL1, CXCL9, and CXCL10, cytokines/chemokines typically associated with lung pathology at 7 dpi (Figure 3B) (P14). These levels of cytokines increased as the mouse age at inoculation increased. P7 mice also had reduced neutrophil recruitment to the lungs compared to older mice at 7 dpi (P14). Mice inoculated at P7 also did not gain as much weight as control mice did, suggesting severe disease [26]. Thus, similarly to human infants, neonatal mice are highly susceptible to RSV infections.

Though there are multiple studies which have observed neonatal mice failing to control RSV infections, there is also evidence that the neonatal response can be stimulated or improved. In experiments by Yamaguchi et al., P6 neonatal mice were pretreated with a TLR9 ligand (CpG) and then inoculated with RSV. Pretreated mice had increased expression of MHCII and CD80 on CD11c cells and IFN-γ production by NK cells at 1 dpi (P7). This suggests that TLR9 stimulation modifies the APC phenotype in the lungs early in infection and may modulate the response to skew away from TH2 [126]. Ruckwardt et al., 2018, also demonstrated that neonatal mice have two unique DC populations that respond to RSV infection [12]. Additionally, neonatal mice immunized with RSV protein had humoral responses with IgG2 antibodies that successfully neutralized the virus. These vaccinated mice also had IFN-γ producing CD4^+^ and CD8^+^ T cells, indicating the development of a TH1 response. However, vaccination of neonatal mice with this other factor induced IgG1 antibody titers associated with a TH2 response [127]. Similarly, infection of neonatal mice with RSV induced RSV-specific antibodies and CD8 T cells responses [128].

As has been previously observed, while neonates appear to have the natural capability to develop effective responses, the TH2 skewing of the neonatal immune response impedes these efforts. As such, P2–P4 neonatal mice inoculated with RSV had low IFN-γ levels as a consequence of reduced alveolar macrophage responses at 7 dpi (P9–P11). These effects, however, were remedied via the administration of IFN-γ [55]. Additionally, the infection of P5 neonatal mice with RSV induced IL-33 expression and an increase in ILC2 numbers in the lungs at 1 dpi (P6), which was not observed in adult mice. Blocking the production of IL-33, however, resulted in reduced RSV immunopathogenesis [62]. Additionally, neonatal AMs made IFN-I upon exposure with RSV; however, IL-10 production from P6 neonatal B cells impedes IFN-I production [78]. Similarly, P4 neonatal antibody production against RSV was found to be limited at 4 dpi (P8); however, the depletion of NK cells significantly increased antibody titers [10]. As RSV remains a major cause of infant illness, extending this work to better understand its virulence in the very young will be important.

### 4.3. Influenza

Influenza viruses are enveloped viruses of the Orthomyxoviridae family. Symptoms include fever, cough, sore throat, fatigue, among others [129]. While all ages are infected, adults typically experience acute, mild symptoms compared to infants [130]. Similarly, infant mice utilized in influenza experiments typically experience more severe disease [28,67]. As such, many experiments aim to assess the neonatal cells and responses which drive this increased susceptibility. To this end, it has been observed that P2 mice inoculated with influenza had neutrophil and eosinophils recruited to the lung, but limited T cell responses, suggesting an impaired TH1 response (Figure 3C) [28]. There is also evidence suggesting an important role for AMs in the neonatal response to influenza. Neonatal neutrophil-derived 12-hete is required for the self-renewal and maintenance of alveolar macrophages, and its depletion resulted in reduced AM populations and greater susceptibility to influenza [17]. Thus, neonatal innate immune responses are an important factor for disrupting viral replication and the progression of disease in the lungs.

In addition to the important role of innate immune cells in the neonatal response to influenza, T cell responses have also been observed to be vital. This is of particular interest due to the presence of T cell subsets that appear to be unique to the neonatal immune system (see above). To this end, P2 neonatal mice inoculated with influenza had reduced infiltration of T cells to the lungs at 6 dpi (P8) and delayed clearance compared to adults. Interestingly, the depletion of Tregs caused a subsequent increase in CD4^+^ T cells in the lungs, though they did not appear to contribute to the clearance of infection. This does suggest, however, that particular T cell subsets are important in regulating neonatal responses to influenza [74]. Another T cell subset which may be important for neonatal influenza infections is γ/δ T cells. As such, the inoculation of P7 neonatal mice with influenza induced the accumulation of IL-17 producing γ/δ cells. Additionally, P7 γ/δ-deficient pups had increased mortality from influenza, suggesting an important role in the neonatal response. The IL-17 γ/δ T cells also promoted the lung infiltration of ILC2s and Tregs, suggesting a role in tissue repair [67]. However, it has also been observed that P2 mice inoculated with influenza had sufficient T cell migration, though it was delayed until 2 weeks pi (P16), along with T cell activation and the expression of TNF-α [28]. This work suggests that T cells play an important role in the neonatal response to influenza and further research is required to better define their contributions.

## 5. Conclusions and Future Directions

The neonatal immune system is a complex network with a primary goal of ensuring the survival of the host via protection against dangerous pathogens. Compared to the well-studied adult immune system, it may appear deficient in some respects, but viewing the neonatal immune system as an incomplete version of the adult’s is missing its critical and unique challenges. The most immediate challenge is the exit from the near-sterile uterus, which represents a unique transition which suddenly bombards the immune system with huge numbers and a diversity of microbes, each expressing thousands of antigens and many of which immediately begin to establish permanent residence in intimate contact with all body surfaces. We have previously promoted the view of the mammalian immune system as a form of microbiome management [131]. From this standpoint, birth initiates a sudden and intense period of rapid assimilation of novel commensals and symbionts. While the adult immune system benefits from prior “knowledge” of these organisms, mostly dealing with a small number of new potential pathogens at any given time, the neonatal one must rapidly and efficiently determine the benefits and risks of reaction to many of each nearly simultaneously. We propose a view of neonatal immunity as not a flawed version of adult immunity, but as a system evolved to face this particular challenge, very different from that of an adult. With this perspective, and a widening array of experimental tools, a deeper understanding of the workings of the neonatal immune system should emerge that will better inform our approaches to protect newborns from disease.

## Data Availability

Not applicable.
